# Rule reversal: Ecogeographical patterns of body size variation in the common treeshrew (Mammalia, Scandentia)

**DOI:** 10.1002/ece3.3682

**Published:** 2018-01-04

**Authors:** Eric J. Sargis, Virginie Millien, Neal Woodman, Link E. Olson

**Affiliations:** ^1^ Department of Anthropology Yale University New Haven CT USA; ^2^ Division of Vertebrate Zoology Yale Peabody Museum of Natural History New Haven CT USA; ^3^ Redpath Museum McGill University Montreal QC Canada; ^4^ United States Geological Survey Patuxent Wildlife Research Center National Museum of Natural History Smithsonian Institution Washington DC USA; ^5^ University of Alaska Museum University of Alaska Fairbanks Fairbanks AK USA

**Keywords:** Bergmann's rule, geographical variation, island area, island rule, latitude, Malay Peninsula, mammals, sea depth, Southeast Asia, *Tupaia glis*

## Abstract

There are a number of ecogeographical “rules” that describe patterns of geographical variation among organisms. The island rule predicts that populations of larger mammals on islands evolve smaller mean body size than their mainland counterparts, whereas smaller‐bodied mammals evolve larger size. Bergmann's rule predicts that populations of a species in colder climates (generally at higher latitudes) have larger mean body sizes than conspecifics in warmer climates (at lower latitudes). These two rules are rarely tested together and neither has been rigorously tested in treeshrews, a clade of small‐bodied mammals in their own order (Scandentia) broadly distributed in mainland Southeast Asia and on islands throughout much of the Sunda Shelf. The common treeshrew, *Tupaia glis*, is an excellent candidate for study and was used to test these two rules simultaneously for the first time in treeshrews. This species is distributed on the Malay Peninsula and several offshore islands east, west, and south of the mainland. Using craniodental dimensions as a proxy for body size, we investigated how island size, distance from the mainland, and maximum sea depth between the mainland and the islands relate to body size of 13 insular *T. glis* populations while also controlling for latitude and correlation among variables. We found a strong negative effect of latitude on body size in the common treeshrew, indicating the inverse of Bergmann's rule. We did not detect any overall difference in body size between the island and mainland populations. However, there was an effect of island area and maximum sea depth on body size among island populations. Although there is a strong latitudinal effect on body size, neither Bergmann's rule nor the island rule applies to the common treeshrew. The results of our analyses demonstrate the necessity of assessing multiple variables simultaneously in studies of ecogeographical rules.

## 1 INTRODUCTION

Intraspecific geographical variation often presents vexing challenges to taxonomists, but such variation is essential for evolution and provides opportunities for insights into its underlying mechanisms. Several environmental factors are known to drive geographical variation in morphology among mammals, including temperature gradients and isolation on islands or island‐like features (e.g., Millien et al., 2006). The magnitude of the effect of these factors on morphological variation may differ across species' traits (e.g., Souto‐Lima & Millien, 2014; Teplitsky & Millien, 2013). The resulting patterns of variation form the basis of a number of ecogeographical “rules” that attempt to describe such patterns and/or infer cause.

The island rule (or Foster's rule) describes a commonly documented pattern in which the body size of large‐bodied mammals on islands is smaller than that of a closely related population on the mainland (dwarfism), whereas small‐bodied mammals tend to be larger on islands than on the mainland (gigantism) (Foster, 1964; Lomolino, 1985, 2005; Lomolino et al., 2013; Millien et al., 2006; Van Valen, 1973). This “rule” has been challenged, however, by several studies (Meiri, Dayan, & Simberloff, 2004, 2005, 2006) arguing that such size variation may vary among taxa (Meiri, Cooper, & Purvis, 2008)—applying, for example, to primates (Bromham & Cardillo, 2007; Welch, 2009) but not carnivorans (Meiri et al., 2004, 2005, 2006)—thereby challenging the notion that such “rules” are in fact universal.

Island area and distance from the mainland—the latter often used as a proxy for the degree of isolation—are also correlated with body size on islands, with greater magnitude of size change expected on smaller, more isolated islands (Heaney, 1978; Lomolino, 2005; Millien, 2011). As with the general island rule, the influence of these factors has been challenged (Meiri et al., 2005, 2006), although variation is expected among different ecomorphs (Lomolino, 2005). For example, island area and isolation appear to be related to size variation in the Asian tri‐colored squirrel (*Callosciurus prevostii*; Heaney, 1978; Lomolino, 2005, figure 3) but not in carnivorans (Meiri et al., 2005, 2006) or long‐tailed macaques (*Macaca fascicularis*; Schillaci, Meijaard, & Clark, 2009).

Bergmann's rule describes the pattern in which species of a genus in colder climates (generally occurring at higher latitudes) have larger mean body sizes than congeners in warmer climates (at lower latitudes), and this rule has been extended to populations within a species (e.g., Ashton, Tracy, & Queiroz, 2000; Bergmann, 1847; Freckleton, Harvey, & Pagel, 2003; Mayr, 1956; Meiri & Dayan, 2003; Millien et al., 2006). Such a trend appears to be generally supported among mammals (Millien et al., 2006: 71% of 149 mammal species), although it is more common in larger‐bodied species (Freckleton et al., 2003; Meiri & Dayan, 2003).

Treeshrews (order Scandentia) are small‐bodied mammals (adults weigh less than 315 g; Sargis, 2002) distributed across much of Southeast Asia, including on numerous islands (see Lyon, 1913; Roberts, Lanier, Sargis, & Olson, 2011). They are seemingly poor overwater dispersers, so their distribution on islands is presumably attributable to vicariance resulting from sea‐level fluctuations in most cases (Olson, Sargis, & Martin, 2005; Roberts et al., 2011). Although they have been included in some taxonomically broad tests of the island rule (e.g., Lomolino et al., 2013; Meiri et al., 2008), they have never been the focus of an intensive study, and recent taxonomic revisions that have greatly altered our understanding of the geographical ranges of individual species (Sargis, Campbell, & Olson, 2014; Sargis, Woodman, Morningstar, Reese, & Olson, 2013; Sargis, Woodman, Morningstar, Reese, & Olson 2014; Sargis, Woodman, Reese, & Olson, 2013) have not been taken into account in these meta‐analyses. Furthermore, Bergmann's rule has never been tested in treeshrews, nor has the possible interaction between these two rules (e.g., Fooden & Albrecht, 1993).

**Figure 1:**
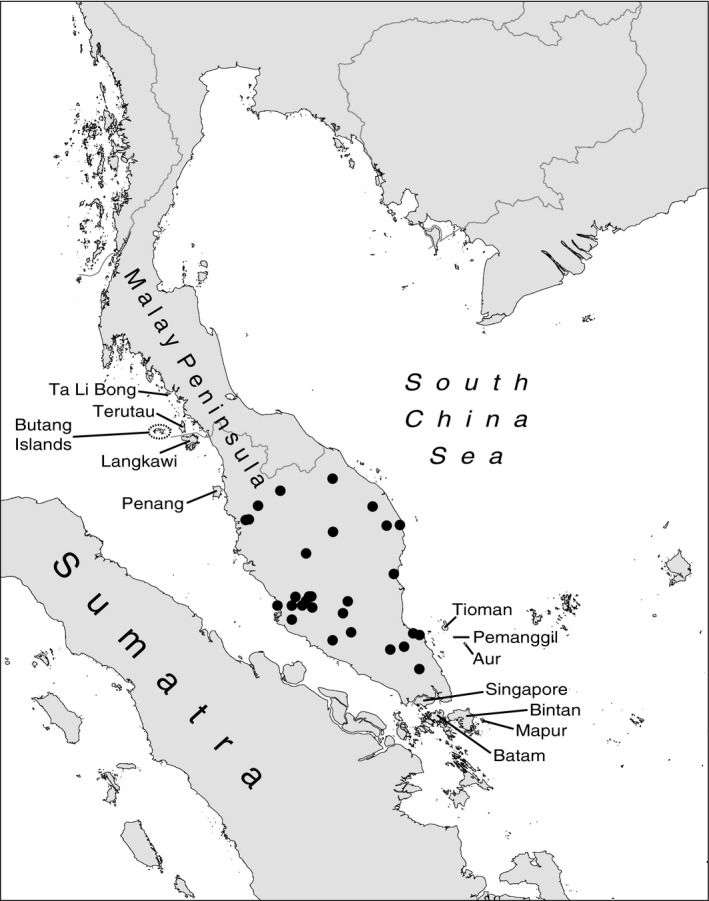
Map of the Malay Peninsula and offshore islands discussed in the text (modified from Sargis et al., 2017). Black circles represent mainland localities (see Appendix S1)

The common treeshrew, *Tupaia glis* (Diard, 1820), is widespread throughout the Malay Peninsula, from about 1.25º to about 10°N latitude, and occurs on a large number of adjacent offshore islands that extend its distribution south to about 0.8°N latitude (Figure 1; Sargis, Woodman, Morningstar, Bell, & Olson, 2017). These are primarily continental islands that were connected to one another and to the mainland during the Pleistocene and became isolated as sea level rose following the Last Glacial Maximum (Voris, 2000). The common treeshrew averages about 152 g (Sargis, 2002), making it a particularly suitable taxon for testing the island rule, as it is between the hypothesized “optimal” mammalian body masses of 100 and 1000 g predicted by Brown, Marquet, and Taper (1993) and Damuth (1993), respectively. As part of our ongoing study of treeshrews, we investigated how island size, maximum modern sea depth between the island and the mainland, and distance from the mainland relate to body size of insular *T. glis* populations, while controlling for the additional effect of latitude on body size in this species.

## 2 MATERIALS AND METHODS

For the purposes of this study, we recognize *Tupaia glis* as comprising those populations taxonomically equated with this species by Helgen (2005), with the exception of several populations we recently recognized as distinct (*T. ferruginea* from Sumatra and Tanahbala Island; *T. discolor* from Bangka Island; *T. hypochrysa* from Java; and *T. chrysogaster* from the Mentawai Islands, including Siberut Island: Sargis, Woodman, Reese, et al., 2013; Sargis, Woodman, Morningstar, et al., 2013; Sargis, Woodman, et al., 2014).

We recorded craniodental measurements from 260 specimens of *Tupaia glis*, collected from the Malay Peninsula (95 specimens) and 13 nearshore islands to the west, east, and south of the Malay Peninsula (165 specimens), including 23 *T. glis* from the type locality on Penang Island (Figure 1). Museum catalog numbers and localities for these specimens are listed in Appendix 1. We also recorded four geographical characteristics for each of our localities (i.e., mainland plus each of the individual islands) that we used as variables in our analyses of ecogeographical variation: (i) Latitude: Localities range from 1.00°N to 7.25°N in latitude; (ii) Island Area: Areas range from 10 to 1173 km^2^; (iii) Distance to Mainland: The minimum distance to the Malay Peninsula varies between 1 and 68 km and contributes to island isolation; and (iv) Sea Depth: The maximum modern sea depth between each island and the mainland ranges from 6 to 81 m and contributes to island isolation, both in terms of amount of time in isolation and current difficulty in crossing because of water barriers (Table 1).

**Table 1:** Latitude (N), Island Area (km^2^), Distance to Mainland (km), and maximum Sea Depth (m) for western, eastern, and southern islands

Island	Latitude	Island Area	Distance to Mainland	Maximum Sea Depth^e^
Western				
Ta Li Bong	7.25	35.00^c^	2.9^b^	6
Penang	5.32–5.47	295.30^a^	5.0^a^	17
Terutau	6.58	151.20^b^	13.0^a^	22
Langkawi	6.23–6.37	363.00^a^	15.0^a^	21
Adang (Butang)	6.55	26.40^b^	53.1^b^	51
Rawi (Butang)	6.55	28.82^b^	58.4^b^	59
Eastern				
Tioman	2.80–2.88	136.00^d^	37.8^b^	46
Pemanggil	2.58	10.32^b^	49.1^b^	46
Aur	2.45	14.62^b^	64.7^b^	68
Southern				
Singapore	1.30–1.34	536.40^a^	1.0^a^	13
Batam	1.08	399.10^a^	15.0^a^	81
Bintan	1.04–1.08	1173.00^a^	20.0^a^	61
Mapur	1.00	27.90^b^	67.5^b^	49

a http://islands.unep.ch/isldir.htm

b Google Earth version 7.0.3.8542

c http://en.wikipedia.org/wiki/Ko_Libong

d http://en.wikipedia.org/wiki/Tioman_Island

e GeoMapApp 3.3.0. (Marine Geoscience Data System. 2012. http://www.geomapapp.org; Ryan et al., 2009)

To evaluate morphological variation within and among populations, EJS recorded 22 craniomandibular variables (Table 2) employed in previous studies of treeshrews (Sargis, Woodman, Morningstar, et al., 2013; Sargis, Woodman, et al., 2014; Sargis, Campbell, et al., 2014; Sargis et al., 2017) from adult skulls (those with fully erupted permanent dentition) using digital calipers. Total length and body weight were recorded from skin tags or the original field notes of collectors. All craniomandibular measurements are in millimeters and were measured to the nearest 0.01 mm; they are tabled in Appendix S1. Our sample includes the holotypes of 10 species or subspecies. Summary statistics of craniomandibular and external variables, including mean, range, 95% confidence interval, standard deviation, coefficient of variation, and percent not available, are presented in Table 3.

**Table 2:** Measurement descriptions (and abbreviations) following Sargis, Woodman, Morningstar, et al. (2013), Sargis, Woodman, et al. (2014), Sargis, Campbell, et al. (2014), Sargis et al. (2017). Uppercase abbreviations for teeth (i.e., I, C, P, M) refer to maxillary and premaxillary teeth; lowercase abbreviations (i, c, p, m) refer to mandibular teeth

Condylopremaxillary length (CPL): greatest distance between rostral surface of premaxilla and caudal surface of occipital condyle. Condyloincisive length (CIL): greatest distance between anterior‐most surface of I1 and caudal surface of occipital condyle. Upper toothrow length (UTL): greatest distance between anterior‐most surface of I1 and posterior‐most surface of M3. Maxillary toothrow length (MTL): greatest distance between anterior‐most surface of C1 and posterior‐most surface of M3. Epipterygoid‐premaxillary length (EPL): greatest distance between rostral surface of premaxilla and caudal surface of epipterygoid process. Palatopremaxillary length (PPL): greatest distance between rostral surface of premaxilla and caudal surface of palatine. Epipterygoid breadth (EB): greatest distance between lateral points of epipterygoid processes. Mastoid breadth (MB): greatest distance between lateral apices of mastoid portion of petrosal. Lacrimal breadth (LB): greatest distance between lateral apices of lacrimal tubercles. Least interorbital breadth (LIB): least distance between the orbits. Zygomatic breadth (ZB): greatest distance between lateral surfaces of zygomatic arch. Braincase breadth (BB): greatest breadth of braincase. Lambdoid‐premaxillary length (LPL): greatest distance between rostral surface of premaxilla and caudal surface of lambdoid crest. Condylonasal length (CNL): greatest distance between rostral surface of nasal and caudal surface of occipital condyle. Postorbital bar‐premaxillary length (PBPL): greatest distance between rostral surface of premaxilla and caudal surface of postorbital bar. Lacrimal tubercle‐premaxillary length (LTPL): greatest distance between rostral surface of premaxilla and caudal surface of lacrimal tubercle. Lambdoid crest height (LCH): greatest distance from apex (or apices if bilobate) of lambdoid crest to both ventral apices of occipital condyles (i.e., along midline). Mandibular height (MH): greatest distance between coronoid and angular processes of mandible. Mandibular condyle height (MCH): greatest distance between mandibular condyle and angular process of mandible. Mandibular condyle width (MCW): greatest distance between medial and lateral surfaces of mandibular condyle. Mandibular condyloincisive length (MCIL): greatest distance between anterior‐most surface of i1 and caudal surface of mandibular condyle. Lower toothrow length (LTL): greatest distance between anterior‐most surface of i1 and posterior‐most surface of m3.

**Table 3:** Summary statistics for the 22 skull measurements. Abbreviations for measurements are defined in Table 2. Statistics are sample size (*n*), mean, range, 95% confidence interval (CI), standard deviation (*SD*), coefficient of variation (CV), and percent not available (%NA)

Measurement	*n*	Mean (mm)	Min (mm)	Max (mm)	95% CI	*SD*	CV	%NA
CPL	224	47.52	43.56	51.57	0.22	1.66	0.03	13.85
CIL	199	46.94	43.16	51.12	0.23	1.65	0.04	23.46
UTL	195	27.06	24.97	29.62	0.13	0.91	0.03	25.00
MTL	207	18.59	17.09	20.90	0.09	0.67	0.04	20.38
EPL	201	34.75	31.75	38.00	0.18	1.26	0.04	22.69
PPL	230	28.69	26.24	31.25	0.13	1.03	0.04	11.54
EB	158	11.48	9.72	13.07	0.10	0.62	0.05	39.23
MB	215	18.04	16.99	19.43	0.07	0.52	0.03	17.31
LB	209	18.81	17.07	20.94	0.11	0.81	0.04	19.62
LIB	242	14.40	12.48	16.46	0.09	0.72	0.05	6.92
ZB	221	25.37	22.76	28.69	0.15	1.14	0.04	15.00
BB	222	19.22	17.79	20.44	0.07	0.54	0.03	14.62
LPL	212	51.03	47.08	55.05	0.23	1.73	0.03	18.46
CNL	226	45.80	41.91	51.32	0.23	1.73	0.04	13.08
PBPL	239	34.80	31.91	37.84	0.16	1.23	0.04	8.08
LTPL	233	23.85	21.08	26.45	0.13	1.03	0.04	10.38
LCH	215	12.41	11.47	13.50	0.06	0.47	0.04	17.31
MH	240	13.60	12.04	15.48	0.09	0.72	0.05	7.69
MCH	247	8.99	7.82	10.48	0.07	0.52	0.06	5.00
MCW	249	3.21	2.50	3.97	0.03	0.25	0.08	4.23
MCIL	222	37.77	34.83	41.00	0.17	1.32	0.03	14.62
LTL	211	25.52	23.77	27.69	0.11	0.82	0.03	18.85
Total length	74	340.36	244.00	395.00	4.74	20.47	0.06	71.54
Body weight^a^	40	132.52^a^	67.00	204.00	11.46	35.83	0.27	84.62

a in grams (g)

The craniomandibular dataset included a significant amount of missing data (15.8%) resulting from damaged or incomplete specimens that would have prevented the statistical analysis of our complete dataset. The missing cases were thus imputed using the “mice” method with predictive mean matching, as described in Clavel, Merceron, and Escarguel (2014). This method uses model estimates to fill in missing values in a dataset with multiple imputations. We performed our analyses on data averaged from 50 independent imputations.

We then performed a principal components analysis (PCA) on the 22 ln‐transformed craniomandibular variables and retained the first component (PC1) for further analyses. Next, we tested for overall sexual dimorphism in PC1 on the complete dataset, with a Welch two‐sample mean comparison test. Sex was included as a variable in all subsequent models as a factor to account for sexual dimorphism.

To test for Bergmann's rule (i.e., a significant positive relationship between body size and latitude) and an island effect on body size simultaneously, we ran a mixed‐effect model on the entire dataset (island and mainland populations) with Latitude, Source of the population (island versus mainland), and Sex as fixed factors, and Locality (13 island localities and mainland) as a random factor. We also tested whether the latitudinal trend differed between island and mainland populations (interaction term between the variables Latitude and Source), and whether sexual dimorphism was different between island and mainland populations (interaction term between the variables Sex and Source). Variance inflation factors for our fixed effects were estimated (checking for values below a threshold of 2.5), and diagnostic plots (residual plots and q‐q plots) were used to evaluate the fit of the model.

We further explored the effect of a number of additional factors on the variation in body size among the 13 island populations. We used Distance to Mainland as a measure of isolation, but also included Sea Depth in this analysis. Sea Depth is relevant here in the context of the paleogeography of the islands and how they formed. Because these islands are the result of sea level rise following the Last Glacial Maximum (Voris, 2000), sea depth between the islands and the mainland can be viewed as a proxy for time since isolation and not simply degree of geographical isolation. We used a hierarchical partitioning analysis to estimate the contribution of Latitude, Island Area, Distance to Mainland, and Sea Depth to the variance in PC1 because all of these factors were significantly correlated with each other (see below). Hierarchical partitioning allows the simultaneous analysis of the effects of multiple correlated factors on a response variable, independently or in combination. Although it can indicate whether a given factor is significantly contributing to the variance in the data, this analysis does not allow estimation of the strength of the effect of each factor. Therefore, we also ran a linear model with PC1 as a response variable and Latitude, Island Area, Distance to Mainland, Sea Depth, and Sex as explanatory variables. Because of collinearity in the explanatory variables, we did not attempt to model or estimate the strength of the main effects, but instead focused on determining the sign of these effects (i.e., positive or negative) because we were interested in testing the generality of ecogeographical patterns.

Finally, to explore shape differentiation among island populations, we performed a canonical variate analysis (CVA) on the 22 ln‐transformed skull variables. For this analysis, we assigned each island to one of three geographical groups (western, eastern, or southern) based on its location relative to the Malay Peninsula (see Table 1, Figure 1; Sargis et al., 2017).

All analyses were performed with the R statistical software version 3.1.0 (R Core Team, 2014), with the packages “MASS” (Venables & Ripley, 2002), “mice” (van Buuren & Groothuis‐Oudshoorn, 2011), “shapes” (Dryden, 2015), “ade4” (Dray & Dufour, 2007), “Morpho” (Schlager, 2015), ellipse (Murdoch & Chow, 2013), and lme4 (Bates, Maechler, Bolker, & Walker, 2015).

## 3 RESULTS

The first component (PC1) from our PCA of skull variables yielded an eigenvalue of 15.62 and explained 71% of the variance in the skull data (Table 4). PC1 was negatively correlated with all 22 craniodental measurements (Table 4), so higher scores represent smaller skull sizes. Both total length and body weight were negatively correlated with PC1 (r = −.46, *p *<* *.0001 and r = −.47, *p *<* *.002, respectively).

**Table 4:** First principal component (PC1) loadings for the 22 skull measurements. Abbreviations for variables are defined in Table 2

Measurement	PC1
LPL	−0.9713
CPL	−0.9704
CIL	−0.9669
PBPL	−0.9614
EPL	−0.9602
MCIL	−0.9588
LTPL	−0.9422
PPL	−0.9383
CNL	−0.9369
UTL	−0.9011
LTL	−0.8747
ZB	−0.8315
MTL	−0.8207
LB	−0.8151
LIB	−0.7635
LCH	−0.7531
MH	−0.7350
MB	−0.7218
MCW	−0.6561
MCH	−0.6518
BB	−0.6401
EB	−0.5567
Eigenvalue	15.624
% variance	71.022

We detected significant sexual dimorphism in PC1 (*t* = 2.40, *p *<* *.0172). Overall, males were larger and had lower PC1 scores than females. Therefore, we included Sex as a factor in all subsequent analyses.

**Figure 2:**
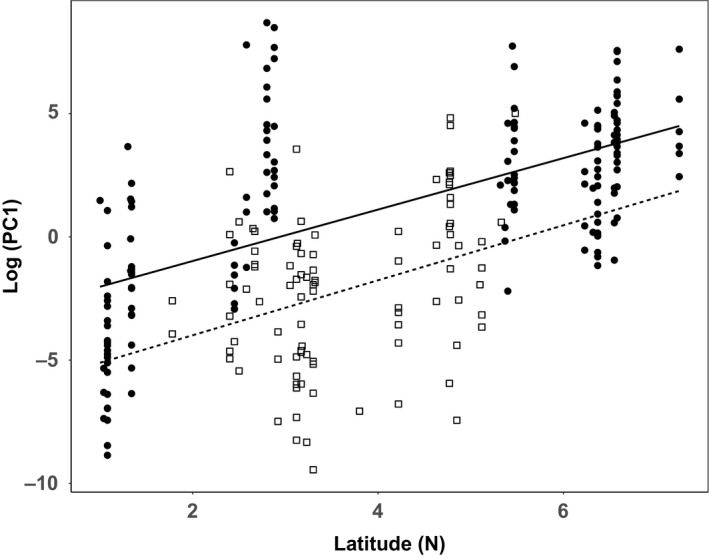
Linear relation between PC1 and latitude among treeshrew individuals from the Malay Peninsula (mainland) and 13 nearshore islands: PC1 increases with latitude; filled circles: islands, open squares: mainland; solid line: linear relation for islands; dotted line: linear relation for mainland; the two regression lines are parallel but differ in elevation

A mixed‐effect model with PC1 as a response variable revealed a significant positive effect of Latitude (*t* = 3.85, *p *<* *.0027; Table 5, Figure 2), indicating decreasing body size with increasing latitude. The strength of this relationship did not differ between islands and the mainland (*p *=* *.79). As already detected with the mean comparison test, males appeared to be larger in body size (smaller PC1) than females (*t* = −2.48, *p *<* *.014) independent of the Source of the population (mainland or islands, *p *=* *.20). There was no overall island effect on body size, as the variable Source (islands versus mainland) did not have any significant effect on PC1 (*p *=* *.44).

**Table 5:** Effect of latitude and the source of the population (island or mainland) on PC1; estimate, standard error (*SE*), degrees of freedom (*df*), *t* statistics (*t* value), and significance level (*p*r[>*t*])

	Estimate	*SE*	*df*	*t* value	*p*r(>*t*)
Latitude	1.03	0.27	11.09	3.85	.0027
Source	−2.16	2.73	12.60	−0.79	.4429
Sex	−0.96	0.39	240.51	−2.48	.0140
Latitude × Source	−0.10	0.39	45.69	−0.27	.7899
Sex × Source	−0.84	0.66	239.17	−1.27	.2044

We further explored the effects of Latitude, Island Area, Distance to Mainland, and Sea Depth on variation in PC1 across the 13 island populations. Because Island Area was correlated with Latitude (r = −.26, *p *<* *.0009), Distance to Mainland (r = −.54, *p *<* *.0001), and Sea Depth (r = −.17, *p *<* *.029), we used a hierarchical partitioning analysis with these four variables and Sex as factors, and PC1 as a response variable. The hierarchical partitioning analysis revealed that Latitude (independent contribution of 41.9%; Z = 25.10, *p *<* *.05) and Sea Depth (independent contribution of 28.7%; Z = 17.48, *p *<* *.05) explained most of the variation in PC1 on islands. Additional variance was explained by Distance to Mainland (14.6%; Z = 8.23, *p *<* *.05) and Island Area (11.4%; Z = 6.40, *p *<* *.05), although not significantly by Sex (3.4%; Z = 1.35, *p *>* *.05) (Table 6). Because we detected significant sexual dimorphism in our data, this latter result suggests that Sex is not contributing to the variance in body size independently, but jointly with other factors. However, the joint contribution in variance cannot be tested for significance in a variance partitioning analysis. A linear model with the four geographical variables and Sex as explanatory factors indicated that PC1 scores for island populations of common treeshrews increased with Latitude and Distance to Mainland (indicating body size decrease) and decreased with Sea Depth and Island Area (indicating body size increase). Hence, common treeshrews exhibited smaller body size on smaller islands, but size variation was mostly driven by a negative latitudinal trend and the sea depth between the mainland and islands, as revealed by the hierarchical partitioning analysis.

**Table 6:** Hierarchical partitioning analysis with the independent contribution to variance of each variable (I obs), the percent variance explained (I %), Z score obtained from 1,000 permutations, and associated significance level; *: *p* < .05, ^ns^: *p* > .05

	I obs	I %	Z score
Latitude	0.22	41.85	25.10*
Maximum Sea Depth	0.15	28.74	17.48*
Distance to Mainland	0.08	14.56	8.23*
Island Area	0.06	11.44	6.40*
Sex	0.02	3.40	1.35 ^ns^

**Figure 3:**
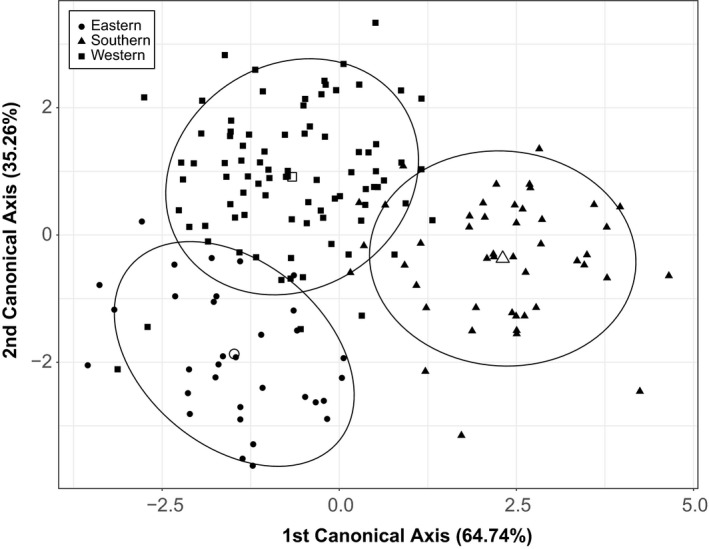
Bivariate plot of the first two axes from canonical variate analysis of the 22 skull variables from the 13 island populations grouped by region as described in Table 1 (see also Figure 1; Sargis et al., 2017); circles: eastern islands, triangles: southern islands, squares: western islands. Open symbols indicate the centroid for each group. All three island groups are significantly distinct from each other (***p < .001)

Our exploration of shape differentiation among the three geographical groups of islands using CVA on the 22 ln‐transformed skull variables yielded clear separation among these island groups. The first two canonical axes explained all of the variance (64.74% and 35.26% for the first and second axes, respectively). Overall classification accuracy for individuals was 91.5%: Four of the 32 individuals from the eastern island group were assigned incorrectly to the western island group; five individuals from the southern islands were incorrectly assigned to the western island group; and five individuals from the western islands were misassigned: four to the eastern island group and one to the southern island group. All three island groups were significantly distinct from each other based on Mahalanobis distances (all *p *<* *.001, 1,000 permutations; Figure 3).

## 4 DISCUSSION

The common treeshrew, *T. glis*, is endemic to Southeast Asia, where it is an abundant and common component of tropical forest mammal assemblages in the region. Although it has featured prominently in biomedical research (Fuchs & Corbach‐Söhle, 2010), it remains poorly studied in the wild. Here, we conducted a review of patterns of morphological variation in the skull of this species to test the validity of two ecogeographical rules that have been described and widely tested in other mammals.

### 4.1 Sexual size dimorphism

Although sexual dimorphism is variably expressed in the coat color of the common treeshrew (Steele, 1983), we found that males had significantly lower PC1 scores and are therefore significantly larger than females. A similar size disparity between the sexes has also been reported in the closely related *T. belangeri* (Collins & Tsang, 1987), demonstrating that sexual size dimorphism warrants consideration in future research on morphological variation in treeshrews.

### 4.2 Bergmann's rule

Our results show that size variation in *Tupaia glis* from the Malay Peninsula and surrounding islands follows a latitudinal gradient, with additional effects of isolation (estimated by minimum distance to the mainland and maximum sea depth between the island and the mainland) and island area in the island populations. In the common treeshrew, body size decreases with increasing latitude, which is the inverse of Bergmann's rule. Although Bergmann's rule is supported by a number of empirical studies in mammals (Ashton et al., 2000; Meiri & Dayan, 2003; Millien et al., 2006), the pattern may be less apparent in temperate large mammals (Steudel, Porter, & Sher, 1994) or subterranean species that live in a more stable environment (Medina, Martí, & Bidau, 2007); however, none of these characteristics applies to the common treeshrew. In their review, Alhajeri and Steppan (2016) detected a weak positive relationship between body mass and temperature among more than 1300 rodent species, but this relationship did not hold when the phylogenetic structure in the data was considered. Instead, larger body mass was related to increasing precipitation (Alhajeri & Steppan, 2016), supporting some previous studies (James, 1970; Yom‐Tov & Geffen, 2006). Alhajeri and Steppan (2016) conducted their study among species within a single mammalian order and concluded that Bergmann's rule may operate within a species (e.g., Ashton et al., 2000; Meiri & Dayan, 2003; Millien et al., 2006) rather than among species within a genus (Bergmann, 1847). Supporting this view, Albrecht (1980) documented Bergmann's rule in the northern pig‐tailed macaque (*Macaca leonina*) but the inverse of Bergmann's rule in the southern pig‐tailed macaque (*M*. *nemestrina*), and Ito, Nishimura, and Takai (2014) found support for Bergmann's rule in the *M. fascicularis* and *M*. *sinica* species groups but not in the *M*. *silenus* or *M*. *sylvanus* groups. Similarly, Ravosa (1998) showed that the common slow loris (*Nycticebus coucang*) follows Bergmann's rule, whereas the pygmy slow loris (*N. pygmaeus*) exhibits the inverse pattern. The mechanisms driving Bergmann's rule are still debated, and any general pattern will be affected by local biotic and abiotic environmental factors, as well as differences in the evolutionary history of study species. Most hypotheses relate body size variation with thermoregulation and/or metabolism (Mayr, 1956), although other factors such as resource availability (McNab, 2010) and levels of competition and predation have been invoked, leading Watt, Mitchell, and Salewski (2010) to designate Bergmann's rule as a “concept cluster.”

### 4.3 Island rule

The single variable most strongly related to body size in our study is latitude. *Tupaia glis* is a generalist, feeding on arthropods, fruits, leaves, seeds, and small vertebrates (Nowak, 1999). With a mean weight of 152 g (Sargis, 2002), the common treeshrew falls well within the range of “small” mammals (Merritt, 2010). For small‐bodied species, the island rule predicts the evolution of larger body size on islands (Foster, 1964; Lomolino, 1985, 2005; Van Valen, 1973). Here, only two of the island populations of common treeshrews exhibit larger body size than on the mainland as predicted by the island rule (e.g., populations from Batam and Bintan islands, both located at the most southern latitude in our study area). However, when we controlled for latitude, none of the populations from the offshore islands around the Malay Peninsula differ in size from the mainland population. The island rule, like Bergmann's rule, may prove to be taxon‐specific in mammals (Meiri et al., 2008), with species within a given order typically following a common pattern. For example, rodent species, with some exceptions, typically evolve larger body size on islands (Durst & Roth, 2015), and patterns found among primates support the island rule as well (Bromham & Cardillo, 2007; Welch, 2009). In contrast, the apparent lack of support for the island rule in the common treeshrew may prove to be the common pattern throughout Scandentia. This contrast is particularly interesting given the close relationship of treeshrews and primates in the supra‐ordinal grouping Euarchonta (e.g., O'Leary et al., 2013). Further testing of the island rule across Scandentia and in Dermoptera (colugos), another euarchontan order that has a similar Southeast Asian distribution, should provide unique insight into these patterns of insular body size variation.

When considering solely insular populations, we found that the secondary driver of *T. glis* body size, after latitude, is maximum sea depth between the mainland and islands: body size of populations on islands separated from the mainland by deeper seas is typically larger. Island area has a tertiary effect on body size: The smaller the island, the smaller the individuals on that island. Hence, common treeshrews are smaller on smaller islands, and the pattern is strongest for populations separated from the Malay Peninsula by shallower seas.

Body size of common treeshrews living on islands is positively correlated with island area, as generally predicted for mammals and other vertebrates (Heaney, 1978; Lomolino, 2005). This correlation of body size with island area was documented by Heaney (1978) for another Southeast Asian mammal, the Asian tri‐colored squirrel (*Callosciurus prevostii*; ~350‐400 g [Thorington, Koprowski, Steele, & Whatton, 2012]). Heaney (1978) predicted that mammals that are slightly smaller than the Asian tri‐colored squirrel, such as the common treeshrew, should increase in body size as island area increases, a prediction supported by our study. This hypothesis may not apply to larger species that are more resource‐limited. Schillaci et al. (2009) found that body size is not related to island area in the long‐tailed macaque (*M. fascicularis*; ~2.5–8.3 kg [Fa, 1989;]) from this region; in fact, long‐tailed macaque populations from Singapore and Bintan (see Table 1 for areas) both exhibit insular dwarfism, possibly related to food limitation and high population density (Fooden & Albrecht, 1993; Schillaci et al., 2007).

Such variation in body size patterns might be expected in a single species distributed among several island groups (see below; Fooden & Albrecht, 1993), especially a species that would fit the “intermediate” category in Heaney's (1978, figure 3) size classification, such as *T. glis*. As Heaney (1978) suggested, mammal species of intermediate size may (i) not vary in their body size pattern, (ii) always converge on the pattern of either a large or small mammal, or (iii) converge on the pattern of either a large or small mammal depending on the conditions. He concluded that the Asian tri‐colored squirrel demonstrated the third option (Heaney, 1978), and this may be the case for the common treeshrew as well, given the assumption of vicariance.

### 4.4 Island group differentiation

The relative effects of island area and isolation acting on body size evolution in island populations of common treeshrews are difficult to tease apart. Factors that could influence body size include the relative timing of establishment on these islands and the different source populations. These factors could account for the clearly distinct morphology among the different island groups (Figure 3), irrespective of island area or degree of isolation. Fooden and Albrecht (1993) demonstrated similar variability among island groups and among islands within island groups in *M. fascicularis* throughout Southeast Asia, where different island populations variably exhibited a decrease, increase, or no change in skull length. Such variation was found both among and within island groups, and Fooden and Albrecht (1993, p. 533) attributed concordance among island populations to “common ancestry, parallel adaptation to local environmental conditions, or coincidence.” These factors may also apply to the variation we found both among and within the western, eastern, and southern island populations in our study (see also Sargis et al., 2017), again, with the assumption of vicariance. Closer comparison of habitat and other conditions on these islands may reveal some critical thresholds in island size that affect the magnitude and direction of change in body size.

## 5 CONCLUSIONS

Ecological factors such as resource limitation, intraspecific/interspecific competition, predation, and parasitism (Heaney, 1978; Lomolino, 2005) are all operating after the establishment of a population on an island. Unfortunately, this history for the islands in our study is not known, but this may have affected the patterns we documented here. Future studies of the phylogeography of *T. glis* and other species on the mainland and offshore islands may provide relevant insight into the establishment of treeshrew populations on these islands as well as the demographic consequences. Furthermore, modern biological surveys of these islands have the potential to provide critical data on variation in species richness and population density that would allow a more thorough assessment of resource availability, ecological release from predation and parasitism, and both inter‐ and intraspecific competition. Finally, our study demonstrates the need for simultaneously testing potentially nonindependent ecogeographical patterns in broadly distributed taxa whose morphology may be influenced by multiple factors, a likely scenario for many species.

## CONFLICT OF INTEREST

None declared.

## AUTHOR CONTRIBUTIONS

E.J.S., V.M., N.W., and L.E.O. conceived the ideas; E.J.S. and L.E.O. collected and curated the data; V.M. analyzed the data; and E.J.S. and V.M. led the writing with considerable input from N.W. and L.E.O.

## APPENDIX 1

### Specimens examined

Specimens of *Tupaia glis* used in this study, organized by sample. As a reference, each sample is accompanied by species or subspecies names [in brackets] with which it has, at times (e.g., Lyon, 1913), been associated. We do not recognize these subspecies as distinguishable units (Sargis et al., 2017). These specimens are housed in the following institutions: The Natural History Museum, London, United Kingdom (BMNH); Field Museum of Natural History, Chicago, IL (FMNH); Muséum national d'Histoire Naturelle, Paris (MNHN); Museum of Vertebrate Zoology at University of California, Berkeley (MVZ); Nationaal Natuurhistorisch Museum, Leiden (RMNH); United States National Museum of Natural History, Smithsonian Institution, Washington, DC (USNM).

***Mainland***

**Peninsular Malaysia** [*T. ferruginea wilkinsoni* Robinson & Kloss, 1911] (*n *=* *95). MALAYSIA: johor: Bekok (USNM 487923, 487925, 487926, 487927, 487928, 487931); Endau River (USNM 112575, 112578, 112580, 112601); Pelepak (BMNH 5.12.7.3; USNM 143268, 143269); Sembrong River (USNM 112616); Tanjong Peniabong (USNM 112658). kedah: Kedah Peak (BMNH 55.1271). kelantan: “K. Pehi Estate” (BMNH 9.5.9.1). negeri sembilan: Ayer Kring (BMNH 55.1252, 55.1254); Gunong Tampin (BMNH 55.1255, 55.1256). pahang: Gunong Tahan (BMNH 6.10.4.10, 6.10.4.11, 55.1219); Punjum, Kuala Lipis (BMNH 55.1233); Rompin River (USNM 115491); Tampong Ubai, Kuantan (BMNH 61.1148); Telom River (BMNH 34.7.18.90, 34.7.18.91, 34.7.18.94, 34.7.18.96, 34.7.18.97, 34.7.18.98, 55.1221); Triang (BMNH 55.1231, 55.1232). perak: Batu Tegor (BMNH 55.1215); Gunong Ijau (BMNH 55.1218); Lenggong (BMNH 55.1213); Maxwell Hill (BMNH 61.1149; USNM 311298); Taiping (BMNH 55.1214); Temengoh (BMNH 55.1217). selangor: Ginting Bidai (BMNH 10.10.1.12, 10.10.1.13, 10.10.1.15, 10.10.1.16, 55.1245, 55.1246, 55.1248); Klang Gates (BMNH 55.1249); Kuala Lumpur (BMNH 34.7.18.99; MVZ 116955, 118599, 183625; USNM 152184, 152185, 487939, 487946); Rawang (BMNH 55.1251, 55.1267); Subang (USNM 487943); Tanjong Duablas (USNM 487940, 487941, 487942); Ulu Gombak (BMNH 61.1150; MNHN 1981‐185); Ulu Langat (USNM 291264; Cheras: BMNH 55.1236, 55.1237, 55.1238, 55.1239, 55.1240, 55.1241; Menuang Gasing: BMNH 55.1243, 55.1244). terengganu: Bukit Besi (USNM 311307, 311308, 311309, 311310, 311311); Kuala Brang (USNM 487949, 487950, 487951, 487953); Tanjong Dungun (USNM 105024, 105025, 105026, 105027, 105030, 105031, 105032, 105033, 105034). THAILAND: trang: Ko‐Khau (BMNH 12.10.7.1—holotype of *T. ferruginea wilkinsoni*).

***Western islands***

**Penang Island** [type locality for *Tupaia glis* (Diard, 1820)](*n *=* *23). malaysia: Penang: Penang Island (BMNH 12.10.7.9, 12.10.7.10, 12.10.7.11, 12.10.7.12, 12.10.7.13, 12.10.7.14, 55.1207, 55.1208, 55.1210, 55.1211, 55.1212, 60.5.4.72, 79.11.21.687; FMNH 98454, 98455, 98456, 98459, 98460, 98462, 98468, 98469, 98470; USNM 487954).

**Butang Islands** [*T. raviana* Lyon, 1911] (*n *=* *8). THAILAND: Satun: Adang Island [=Ko Adang] (BMNH 12.10.22.5, 12.10.22.6, 55.1379, 55.1380; USNM 104354); Rawi Island (BMNH 12.10.22.4, 55.1378; USNM 104355—holotype of *T. raviana*).

**Langkawi and Terutau Islands** [*T. lacernata* Thomas & Wroughton, 1909] (*n *=* *49). MALAYSIA: Kedah: Langkawi Island (BMNH 9.11.1.22, 9.11.1.23, 9.11.1.24, 9.11.1.25, 9.11.1.26, 9.11.1.27, 9.11.1.28, 9.11.1.29, 9.11.1.30—holotype of *T. lacernata*, 55.1381, 55.1382, 55.1383, 55.1384, 55.1385, 55.1386, 55.1387, 55.1389, 55.1390, 55.1391, 55.1392, 55.1393; USNM 104353, 123901, 311302, 311303, 311306). THAILAND: Satun: Terutau Island (BMNH 9.11.1.14, 9.11.1.15, 9.11.1.16, 9.11.1.17, 9.11.1.18, 9.11.1.19, 9.11.1.20, 55.1394, 55.1395, 55.1396, 55.1397, 55.1398, 55.1399, 55.1400, 55.1401, 55.1402, 55.1403; FMNH 43836; USNM 123981, 123982, 123985, 123987, 123988).

**Ta Li Bong Island** [*T. g. umbratilis* Chasen, 1940] (*n *=* *6). THAILAND: Trang: “Telibon” [= Ta Li Bong] Island (BMNH 47.1496—holotype of *T. g. umbratilis*, 55.1374, 55.1375, 55.1376, 55.1377; USNM 83256).

***Eastern islands***

**Aur Island** [*T. pulonis* Miller, 1903] (*n *=* *7). MALAYSIA: Johor: “Aor” (= Aur) Island (BMNH 12.10.22.2, 12.10.22.3, 55.1352, 55.1353, 55.1354, 55.1355; USNM 112449—holotype of *T. pulonis*).

**Pemanggil Island** [*T. pemangilis* Lyon, 1911] (*n *=* *4). MALAYSIA: Johor: Pemanggil Island (BMNH 12.10.22.1, 55.1350, 55.1351; USNM 112499—holotype of *T. pemangilis*).

**Tioman Island** [*T. sordida* Miller, 1900] (*n *=* *22). MALAYSIA: Pahang: Pekan District, Tioman Island (USNM 101746, 101747—holotype of *T. sordida*, 104973, 104974, 104975, 104976), Kampong Tekek (USNM 487932, 487933, 487937, 487938), Kampong Ayer Padi (USNM 487934, 487936), Juara Bay (BMNH 8.1.25.4, 10.10.1.11, 55.1340, 55.1341, 55.1342, 55.1343, 55.1345, 55.1346, 55.1347, 55.1348).

***Southern islands***

**Batam Island** [*T. ferruginea batamana* Lyon, 1907] (*n *=* *17). INDONESIA: “Rhio” (=Riau) Archipelago; “Battam” (=Batam) Island; Senimba Bay (USNM 142151—holotype of *T. ferruginea batamana*, 142152, 143252, 143253, 143254, 143255, 143256, 143257); Tanjong Turut (BMNH 9.4.1.114, 9.4.1.115, 9.4.1.116, 9.4.1.117, 9.4.1.118, 9.4.1.119, 9.4.1.120; RMNH 36091, 36092)

**Bintan Island** [*T. castanea* Miller, 1903] (*n *=* *8). INDONESIA: “Rhio” (=Riau) Archipelago; “Bintang” (=Bintan) Island (USNM 115607, 115608—holotype of *T. castanea*); Sungei Biru (BMNH 9.4.1.101; RMNH 36093, 36094); Pasir Panjang (BMNH 9.4.1.102, 9.4.1.103, 9.4.1.104).

**Mapur Island** [*T. castanea redacta* Robinson, 1916] (*n *=* *1). INDONESIA: “Rhio” (=Riau) Archipelago; “Mapor” (=Mapur) Island (BMNH 26.10.19.4—holotype of *T. castanea redacta*).

**Singapore** [*T. glis*] (*n *=* *20). singapore (BMNH 9.4.1.105, 9.4.1.106, 9.4.1.107, 9.4.1.108, 9.4.1.109, 9.4.1.110, 9.4.1.111, 55.1257, 55.1258, 55.1260, 55.1261, 55.1262, 55.1263, 55.1264, 55.1265, 55.1266; USNM 105078, 105079, 105080, 124317).
